# Contribution of skeletal muscle and serum lipids to muscle contraction induced by neuromuscular electrical stimulation in older individuals

**DOI:** 10.14814/phy2.15236

**Published:** 2022-03-21

**Authors:** Akito Yoshiko, Hisashi Maeda, Hideyuki Takahashi, Teruhiko Koike, Noriko Tanaka, Hiroshi Akima

**Affiliations:** ^1^ Faculty of Liberal Arts and Sciences Chukyo University Toyota Aichi Japan; ^2^ Graduate School of Medicine Nagoya University Nagoya Aichi Japan; ^3^ Faculty of Health and Sport Sciences University of Tsukuba Tsukuba Ibaraki Japan; ^4^ Research Center of Health, Physical Fitness & Sports Nagoya University Nagoya Aichi Japan; ^5^ Graduate School of Education and Human Development Nagoya University Nagoya Aichi Japan

**Keywords:** blood lipids, extramyocellular lipid, intramyocellular lipid, neuromuscular electrical stimulation, older individuals

## Abstract

Intramyocellular lipids (IMCL) stored in droplets in muscle cells and free fatty acids (FFA) from fat cells in the blood are the main substrates of adenosine triphosphate during continuous muscle contractions of relatively lower intensity. Although it is known that the lipid oxidative capacity decreases with aging, the effect of IMCL and FFA on muscle contraction in older individuals remains unclear. The purpose of this study was to investigate the contribution of skeletal muscle lipids and blood lipids as energy sources for muscle contraction in older individuals. Eighteen older individuals (mean age: 70.4 ± 3.5 years) underwent muscle contraction intervention induced by intermittent neuromuscular electrical stimulation (NMES) to the vastus lateralis for 30 min. Fasting blood samples were obtained and proton magnetic resonance spectroscopy (^1^H‐MRS) was performed before and after NMES, and the parameters (including IMCL and extramyocellular lipid [EMCL]) from ^1^H‐MRS, along with FFA and adiponectin levels, were analyzed using the blood samples of all participants. Levels of IMCL and EMCL did not change (*p* > 0.05); however, FFA and adiponectin levels decreased from 1.1 ± 0.5 mEq/L to 0.8 ± 0.2 mEq/L and 12.0 ± 5.3 μg/ml to 11.4 ± 5.0 μg/ml, after NMES (*p* < 0.05), respectively. These findings indicate that serum lipids, but not skeletal muscle lipids, are the energy substrate utilized during involuntary muscle contraction in older individuals.

## Graphical abstract

FFA significantly decreased after muscle contraction, although IMCL and EMCL did not change. These findings suggest that FFA were the energy source utilized during involuntary muscle contraction.

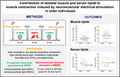

## 1 INTRODUCTION

Lipids and carbohydrates are the principal substrates in adenosine triphosphate (ATP) resynthesis during continuous contractions of skeletal muscle at lower intensity. Previous studies have shown that the energy expenditure during exercise of low to moderate intensity (~50% $\dot{V}$O_2max_) was fueled by free fatty acid (FFA) secreted from adipose tissue (Brooks, 1997; Hawley, 2002). Furthermore, other lipid sources, such as intramyocellular lipid (IMCL), extramyocellular lipid (EMCL), and lipoprotein‐derived triglycerides, have also been identified as energy substrates (Hargreaves & Spriet, 2020). Several studies, using proton magnetic resonance spectroscopy (^1^H‐MRS) and histochemical analysis, have confirmed IMCL in vivo, and showed that the IMCL content was decreased in active muscles after exercise (Harber et al., 2008; Koopman et al., 2006; White et al., 2003). These findings indicate that IMCL is an energy source during exercise.

Aging alters the skeletal metabolic system and is often accompanied by morphological changes. One important morphological change is sarcopenia, which is characterized by a decline in muscle size. Sarcopenia eventually leads to mobility impairment, falls, and physical frailty in older individuals (Cruz‐Jentoft et al., 2019). In addition, the metabolic capacity of the skeletal muscles also decreases with sarcopenia. Decreased mitochondrial oxidative capacity and/or a decreased number of mitochondria induce lower ATP production in the skeletal muscle of older individuals compared with that of young persons (Crane et al., 2010; Karakelides et al., 2010). These age‐related morphological and metabolic changes eventually increase the IMCL content in skeletal muscle (Crane et al., 2010; Petersen et al., 2003). The concentration of IMCL has been implicated in the development of the metabolic syndrome; high IMCL in older individuals relates to the risk of insulin resistance (Coen & Goodpaster, 2012). Hence, understanding the mechanism of IMCL accumulation and reduction would promote the health of older individuals by avoiding and preventing type 2 diabetes.

Physical activity and exercise enhance skeletal muscle lipid metabolism when accompanied by energy expenditure. Endurance exercise (cycling and running) has been shown to enhance skeletal muscle metabolism and reduce IMCL content in activated muscles (Bucher et al., 2014; Krssak et al., 2000; White et al., 2003). In addition, previous studies have found that resistance exercise also enhances lipid metabolism in the skeletal muscle (Creer et al., 2005; Essén‐Gustavsson & Tesch, 1990; Harber et al., 2008; Koopman et al., 2006). Koopman et al. (2006) investigated the effect of resistance exercise (i.e., 10 reps ×8 sets at 75% of the one‐repetition maximum) on IMCL using leg press and leg extension. They showed a 27% decline in muscle lipid content after the resistance exercise. Similarly, it is well known that muscle contraction with dynamic knee extension enhanced FFA uptake in the quadriceps muscles (Laaksonen et al., 2013). These findings imply that IMCL and FFAs contribute to the energy during muscle contraction. As mentioned above, a higher IMCL content has been implicated in the development of the metabolic syndrome (Coen & Goodpaster, 2012); hence, if muscle contraction reduces IMCL concentration in older individuals, our findings would help establish interventions against the incremental risk of age‐related insulin resistance. However, no studies have investigated the effect of muscle contraction on IMCL and FFAs in older individuals.

The quadricep femoris (QF) muscles are often selected to investigate the change in IMCL following resistance exercise because of their importance in locomotive function and activities of daily living (Harber et al., 2008; Koopman et al., 2006). For instance, Harber et al. (2008) utilized a repeated voluntary knee extension task at ~70% of the one‐repetition maximum to observe a 42% decline in IMCL content in the vastus lateralis (VL). This simple task has been used to observe muscle activation and metabolic change in previous studies; however, voluntary knee extension evokes muscle contraction of the rectus femoris, vastus intermedius, and vastus medialis, in addition to the VL, implying that the task would induce the enhancement of lipid metabolism in the four muscles of the QF. The contribution ratio of muscle contraction among the QF muscles is heterogenous and sensitive to change depending on the intensity and knee joint angle (Saito & Akima, 2013); hence, it was unclear how much the target muscle was contracted by the voluntary knee extension task. On the other hand, neuromuscular electrical stimulation (NMES) has been used to induce involuntary contraction of muscle located under the electrode (Akima et al., 2002). With this technique, it is possible to match the location of IMCL measurement with that of the contracted muscle. NMES is also used to increase muscle mass and improve function in the rehabilitation and medical care for the patients with spinal cord injuries, diabetes, or physical disabilities, in addition to that of older individuals. Furthermore, the NMES technique is effective for skeletal muscle metabolism improvement (Gorgey et al., 2012; Hamada et al., 2003). One study has reported that NMES improved insulin sensitivity related to the concentration of IMCL (Joubert et al., 2015). Therefore, we speculated that the mechanical contraction induced by NMES would enhance lipid availability for acute energy demand, eventually decreasing the IMCL store in the target muscle; however, studies on the acute metabolic demands of NMES are scarce.

The purpose of this study was to investigate the contribution of blood lipids, such as FFA, and skeletal muscle lipids (e.g., IMCL and EMCL) to muscle contraction induced by NMES in older individuals. We hypothesized that blood and skeletal muscle lipids would decrease after the muscle contraction due to energy expenditure.

## 2 METHODS

### 2.1 Ethics approval

Ethics approval for this study in humans was granted by the ethics committee of Chukyo University (approval no. 2018‐044) and the Institutional Review Board of Nagoya University, Graduate School of Medicine (approval no. 2019‐0010). All experimental procedures conformed to the *Declaration of Helsinki*, except for registration in a database. Before the experiment, the purpose, procedures, and risks associated with this study were explained to each participant, and written informed consent was obtained from all participants. Participants approved all examination protocols.

### 2.2 Participants

**FIGURE 1:**
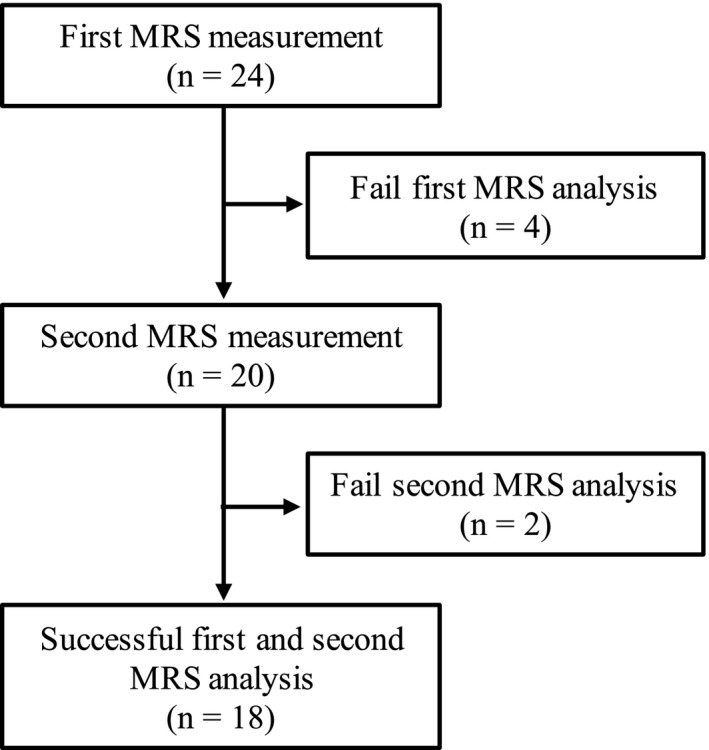
Flow diagram of participants through each stage of this study. Abbreviations: MRS, proton magnetic resonance spectroscopy

We recruited older participants who lived near our university. The inclusion criterion was age ≥65 years. Exclusion criteria were as follows: dependence on others for activities of daily living; diagnosed with impairment of cognitive function and/or dementia by a medical doctor; musculoskeletal, neuromuscular, orthopedic, or cardiovascular diseases; exercise, sports, or physical activities limited by a medical doctor, or has a contraindication to magnetic resonance imaging (e.g., claustrophobia, metallic artificial joint, or heart pacemaker). Twenty‐four men and women participated in this study. The sample size was determined based on previous studies that investigated the effect of acute exercise on IMCL using ^1^H‐MRS (Bucher et al., 2014; Schrauwen‐Hinderling et al., 2003; White et al., 2003). In six participants, the ^1^H‐ MRS analysis failed; thus, IMCL and EMCL data could not be obtained for these participants. Finally, the skeletal muscle lipid content of the 18 participants was successfully obtained (Figure 1).

### 2.3 Experimental design

Participants visited the laboratory on two different days at least 48 h apart. On the first day, we explained the flow of the experiments and the aims of our study. After obtaining each participant's consent, we measured their height and other physical characteristics (e.g., body mass, muscle mass, and body fat) using an analog height meter and bioelectrical impedance analysis (InBody 430; InBody Japan). The InBody 430 body fat analyzer measures impedance across the legs, arms, and trunk via frequencies of 5, 50, and 250 kHz. The system's eight electrodes are located in the footpads mounted on the surface of a platform scale and in the handheld pads extending from the machine's body. Participants were instructed to stand on the electrode plate while squeezing the handle with both hands for 1 or 2 min. The machine automatically measured the impedance and body mass after the participant's height and age were manually entered into the system. Subsequently, the participants practiced maximal voluntary contraction (MVC) using an isometric knee extension task to avoid the learning effect. After the MVC practice, we set electrodes on the surface of the VL, and set the voltage so that it evoked 10%–15% of the MVC force. After the first day, we monitored the participants’ daily level of physical activity using an active monitor. Participants were instructed to wear an activity monitor (HJA‐750C, Omron) at the waist all day (except during bathing and sleeping) for 14 days. The participants were asked to continue their normal activities of daily living during the measurement period. We determined the average amount of time spent in each respective activity intensity [e.g., light intensity: 1.0–2.9 metabolic equivalents (METs), moderate intensity: 3.0–6.0 METs, and vigorous intensity: >6.0 METs]. We asked the participants to avoid impulsive exercise for daily living. Participants were also requested to avoid vigorous exercise, even if it formed part of their normal activities of daily living, in order to exclude the effect of fatigue for 3 days prior to the second day. After 14 days, participants returned their activity monitors by mail. On the second day, ^1^H‐MRS and blood samples were evaluated for all participants. The measurements were performed in the morning (from 9 a.m. to 12 p.m), and participants were instructed to avoid taking any food and drink except water after 12 midnight. After the first blood sampling, ^1^H‐MRS was performed to measure IMCL and EMCL in the VL. Blood samples were collected twice by a licensed clinician. After the first ^1^H‐MRS, participants moved to the experimental chair and the first MVC was measured. All participants underwent stimulation for 30 min while sitting with ankle joints in a fixed position. Immediately after 30 min electrical stimulation, the second MVC and second ^1^H‐MRS values were obtained. Finally, the second blood sampling was obtained from all participants.

### 2.4 MVC measurement

The maximal voluntary isometric knee extension strength of the right leg was measured using a custom‐made dynamometer (Takei Scientific Instruments Co., Ltd.) with a force transducer (LU‐100KSE; Kyowa Electronic Instruments). The hip was fixed to the dynamometer by a strap, and the knee joint angle was fixed at 90° (0° is fully extended). Participants performed a MVC from baseline to maximal effort within 3–4 s and then sustained the contraction for a maximum of 3 s. Two MVC trials were performed and the maximum force generated was selected. The force signals were sampled with a frequency of 1000 Hz through an analog‐to‐digital convertor (PowerLab; ADInstruments), and data were stored in a computer (Mac Book Pro; Apple Inc.). The maximal torque was calculated as the exerted force (N) × the lever arm length of the dynamometer (m).

### 2.5 NMES

Participants were positioned on the measurement chair with the knee joint angle fixed at 90°. The ankle joint was attached to a bar linked to a force transducer. Transcutaneous electrical stimulation (Theratouch 4.7, RICH‐MAR) was applied to the lateral aspect of the QF, on the VL as described previously (Akima et al., 2002). Voltage was delivered via two 5.0 × 9.0 cm electrodes (PALS, Axelgaard Manufacturing Co., Ltd.) applied to the skin, and it was changed as needed to generate a 10%–15% MVC in knee extension. The average voltage was 65 ± 11 mA (range: 40–75 mA). One of the electrode pads were placed 4–6 cm proximal to the superior aspect of the patellar over the VL, and the other was placed 7–12 cm distal to the greater trochanter over the VL. Stimulation consisted of 5‐s trains of 200‐μs biphasic pulses (50‐μs delay) delivered at 50 Hz with a 5‐s on‐ and 3‐s off‐duty cycle for 30 min (total number of stimulations: 225; total stimulation time: 19 min). Knee extension strength was continually monitored during NMES.

### 2.6 ^1^H‐MRS

The ^1^H‐MRS procedure has been previously reported (Akima et al., 2016). Briefly, ^1^H‐MRS measures IMCL of the right thigh via a 4‐channel flex coil (366 ×174 mm). Carefully avoiding visible vascular structures, adipose tissue deposits, and connective tissue, voxels were positioned in the VL at the mid‐thigh between the greater trochanter and lateral condyle of the femur to match the position of the magnetic resonance imaging measurement. The voxel size was dependent on the participant's muscle size (10 × 10 × 10–14 × 14 × 10 mm), but thickness was fixed at 10 mm. ^1^H‐MRS spectra from regions of interest were acquired using a point‐resolved spectroscopy sequence with the following acquisition parameters: TR/TE 4000/30 ms; 128 averages (Boesch et al., 2006). The unsuppressed water signal was subsequently measured in the same voxel under the same shimming conditions. ^1^H‐MRS data were analyzed using LCModel (v.6.2‐4A, Stephen Provencher, Inc.). The spectroscopic data taken from the MR scanner were collected in a computer, and metabolism was quantified using eddy current correction and water scaling. The concentration of water was assumed to be equal to 42.4 mmol/kg wet weight based on a mean adult muscle tissue water content of 77% (Sjøgaard & Saltin, 1982). Concentrations of IMCL (−CH2) and EMCL (−CH2) were collected for the T1 and T2 relaxation effects of the unsuppressed water peak using LCModel control parameter atth2o, which were determined using the following equation: exp. (−TE/T2) [1 − ext. (−TR/T1)] (Drost et al., 2002), assuming relaxation time T1 = 369 ms and T2 = 89.4 ms for the IMCL_CH2_, and T1 = 369 ms and T2 = 77.6 ms for the EMCL_CH2_ (Krssák et al., 2004). The concentration of lipid molecules (total lipid content) was computed by the summation of the IMCL_CH2_ and EMCL_CH2_ concentrations and divided by 31. The value 31 follows from the assumption that the average number of methylene protons is 62 per triglyceride molecule (equivalent to 31 CH_2_ groups) (Boesch et al., 1999; Szczepaniak et al., 1999; Weis et al., 2009). One scan took approximately 30 min.

### 2.7 Analysis of blood lipids

Fasting blood samples were obtained before and after the ^1^H‐MRS. Samples were sent to a commercial laboratory for analysis (BML, Inc.) and serum total cholesterol, low‐density lipoprotein cholesterol, high‐density lipoprotein cholesterol, triglyceride (TG), FFAs, glucose, and adiponectin values were determined.

### 2.8 Statistics

All values are reported as the mean and standard deviation. First, we performed a Kolmogorov–Smirnov test to confirm data normality. The Wilcoxon test was used to compare differences before and after the NMES. Effect size (ES) statistics are provided where appropriate, using *r* for Wilcoxon test. The level of significance was set at *p* < 0.05. All statistical analyses were performed using SPSS Statistics version 22.0J (IBM Japan).

## 3 RESULTS

Age, physical characteristics, and daily physical activity levels at baseline are shown in Table 1. No statistically significant differences were found in knee extension MVC torque before and after NMES (before: 94.3 ± 27.4 Nm; after: 92.0 ± 26.2 Nm; *p* = 0.47; ES [*r*] = 0.16). NMES evoked knee extension strength during stimulation (10.3 ± 4.4 Nm; 11.2 ± 5.9% MVC).

**TABLE 1:** Age, physical characteristics and daily physical activities in all participants (*n* = 18)

Age	70.4 ± 3.5
Sex (men/women)	5/13
Height (cm)	158.1 ± 9.0
Body mass (kg)	56.2 ± 9.7
BMI (kg/m^2^)	22.4 ± 2.6
Whole‐body muscle mass (kg)	21.9 ± 4.6
Fat mass (kg)	15.6 ± 4.9
Fat ratio (%)	27.7 ± 6.7
Daily steps (counts/day)	6022 ± 2190
Rest and light intensity activity (min/day)	743.1 ± 97.3
Moderate intensity activity (min/day)	77.8 ± 24.8
Vigorous intensity activity (min/day)	1.3 ± 1.5

Note

Values represent the mean ± SD.

**FIGURE 2:**
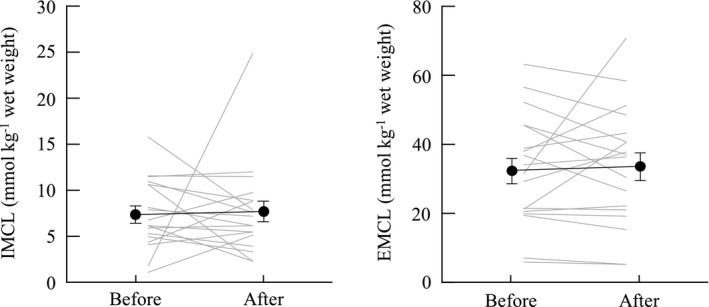
Change in intramyocellular lipids (IMCL) and extramyocellular lipids (EMCL) for the neuromuscular electrical stimulation. Error bars show standard error

IMCL and EMCL content did not change following 30 min of NMES (*p* = 0.50–0.81, ES (*r*) = 0.06−0.16, Figure 2). Total cholesterol, high‐density lipoprotein cholesterol, low‐density lipoprotein cholesterol, TG, FFA, glucose,and adiponectin significantly decreased after NMES (*p* = 0.002 or <0.001, ES (*r*) = 0.72–0.82, Table 2).

**TABLE 2:** Blood lipids, glucose and adipocytokines at before and after the neuromuscular electrical stimulation

	Before (*n* = 18)	After (*n* = 18)	*p*‐value
Total cholesterol (mg/dL)	222.9 ± 29.8	214.5 ± 25.1	0.002
HDL (mg/dl)	66.6 ± 14.0	64.0 ± 14.8	<0.001
LDL (mg/dl)	140.8 ± 31.2	135.4 ± 28.2	<0.001
TG (mg/dl)	107.2 ± 51.5	98.5 ± 45.6	<0.001
FFA (mEq/L)	1.1 ± 0.5	0.8 ± 0.2	0.002
Glucose (mg/dl)	95.1 ± 16.9	83.8 ± 23.2	<0.001
Adiponectin (μg/ml)	12.0 ± 5.3	11.4 ± 5.0	<0.001

Note

Values are the mean ± SD.

Abbreviations: FFA, free fatty achid; HDL, high‐density lipoprotein; LDL, low‐density lipoprotein; TG, triglyceride

## 4 DISCUSSION

We investigated the effect of muscle contraction on skeletal muscle lipids, serum lipids such as FFAs and TG, and glucose in older individuals. Although all serum lipids and glucose decreased after NMES, IMCL, and EMCL did not change. These findings indicate that while serum lipids might be an energy source of involuntary muscle contraction, skeletal muscle lipids do not recruit a substrate for repetitive muscle contractions. This information may be useful for the development of an effective NMES strategy to prevent and improve metabolic impairment in older individuals with a high risk for metabolic disorders.

Regarding the effect of repetitive muscle contractions on serum and muscle lipid profiles, we found that serum FFAs significantly decreased after intermittent muscle contractions (Table 2). According to recent studies, the main source of fat for skeletal muscle oxidation at low exercise intensities in trained men is plasma FFA derived from adipose tissue lipolysis (Hargreaves & Spriet, 2020; Hawley, 2002). Several studies concluded that FFA contributed 25% to 31% of the total energy expenditure when exercising at 55% of the maximal workload intensity (van Loon et al., 2001). Interestingly, the change of plasma FFA during and after endurance exercise has been reported; for instance, Schrauwen‐Hinderling et al. (2003) showed that FFAs increased seventeen‐fold after cycling exercise. This result implies that the increase in FFAs immediately after the exercise is accompanied by the demand of energy substrate from adipose tissue. On the other hand, limited studies have investigated substrate to energy expenditure during resistance exercise. Koopman et al. (2006) showed the effect of resistance exercise on plasma FFAs. They reported that approximately 30% of FFAs declined during 30‐ and 60‐min resistance exercises, but immediately increased after the end of exercise. This result showed that the timing of measurement would be important to understand the dynamic depletion and recovery of FFAs. We obtained a second blood sample at least 30 min after the NMES session instead of immediately after; nevertheless, we still found a 29% decline in serum FFAs compared with the values prior to exercise (Table 2). Considering that our participants were older individuals, it was suggested that age‐related decline in lipolysis contributed to lower values of FFAs 30 min after the exercise (Gao et al., 2020). It has been reported that increased FFA uptake after resistance exercise, and the induction FFA metabolism, continues for some time after exercise (Krssak et al., 2000; Laaksonen et al., 2013). The decline in FFAs after muscle contraction, as shown in our study, indicates FFA uptake in the activated muscle. However, we only obtained blood samples just before and 30 min after muscle contraction, and did not obtain subsequent FFA levels post‐exercise. Furthermore, it should be considered that plasma FFA could show the balance between the metabolism from the skeletal muscle and the supply from the adipose tissue, and they could not be adequately separated. Measuring the post‐exercise effect on FFAs would provide important insights into understanding whole‐body and muscle fat metabolism and supply in older individuals.

IMCL is known as one of the energy substrates for muscle contraction during exercise. Many studies have shown the dynamic decline of IMCL before and after endurance and resistance exercises (Creer et al., 2005; Essén‐Gustavsson & Tesch, 1990; Harber et al., 2008; Koopman et al., 2006). Consistent with these previous studies, we hypothesized that IMCL would decrease after intermittent muscle contraction. Contrary to our hypothesis, we did not observe a change in IMCL after the 30‐min muscle contraction (Figure 2). There are two main reasons for this discrepancy. First, we used NMES to stimulate muscle contraction because we could measure contraction of the target muscle with certainty. It has been shown that the T2 value obtained by magnetic resonance imaging changed after the 30 min muscle contraction in the VL, implying that electrical stimulation only induced contraction of the muscle located under the electrode (Akima et al., 2002). We confirmed the 11.2% ± 5.9% MVC during stimulation for only the VL; therefore, the VL muscle contraction would certainly be derived by NMES in our experiment. It is well known that IMCL is mainly located in type I muscles in close proximity to the mitochondria (van Loon & Goodpaster, 2006). This is understandable as IMCL can be easily assessed as an energy substrate when activating type I muscle fibers. It is well known that the recruitment pattern of the motor unit is different between NMES and voluntary exercise. The Henneman size principle describes progressive motor unit recruitment during voluntary force production, in which slow‐twitch fibers are activated first and fast‐twitch fibers are recruited only during near‐maximal effort (Henneman et al., 1965). On the other hand, NMES recruits motor units in a nonselective activation pattern, in which slow‐twitch and fast‐twitch muscle fibers are activated simultaneously (Gregory & Bickel, 2005). Koopman et al. (2006) reported that lipid content in type I fibers decreased after leg press and leg extension exercises were performed at 75% of maximum effort. It was suggested that almost all type I fibers were recruited for voluntary force production. On the other hand, small forces (e.g., approximately 10% of maximum force) evoked by NMES were generated using both type I and II fibers in our study. Therefore, the absolute contribution of type I fibers during NMES is likely smaller than its contribution during voluntary force production, which may be one of the reasons that the IMCL levels did not change after NMES. Excessive accumulation of IMCL with decreased mitochondrial function is associated with insulin resistance, which eventually leads to type 2 diabetes (Coen & Goodpaster, 2012). Interestingly, Joubert et al. (2015) showed that insulin sensitivity was significantly improved in patients with type 2 diabetes after 1‐week of NMES training. Although the underlying mechanism remains unknown, it is clear from our results that IMCL does not contribute to this improvement. Although many studies have investigated the effect of NMES on muscle mass and strength, information on the effect of muscle lipid metabolism remained scarce. NMES leads to involuntary muscle contraction; hence, this technique is applicable not only in healthy older individuals, but also in older individuals with disabilities and patients lacking motor function control, such as those with spinal cord injuries, paralysis, and stroke. Considering that our study only investigated the acute effect of muscle contraction on IMCL accumulation in healthy older individuals, more studies employing NMES exercises and training with varying durations and intensities should be conducted in varying conditions.

The second reason for the discrepancy in our results was that the participants in our study were older (65–77 years of age). Aging changes muscle metabolism via alterations in mitochondrial content and enzyme activity (Crane et al., 2010; Petersen et al., 2003). Sial et al. (1996) investigated fat and carbohydrate oxidation during endurance exercise in young and older individuals. They found that the proportion of fat oxidation was significantly lower in older individuals compared with that of their young counterparts. Furthermore, with aging, the relative contribution of IMCL as an energy source shifted to FFAs (IMCL contribution was 71.9% in young and 38.7% in old); in other words, older individuals mainly use FFAs as the energy substrate in lipid metabolism (Chee et al., 2016). These findings would explain why FFAs, and not IMCL, significantly changed after exercise in our study (Figure 2 and Table 2). Other methods to decrease IMCL in older individuals should be investigated, considering that NMES had little impact on IMCL content in this study.

We investigated whether EMCL, which is the adipose tissue stored around muscle cells, would change with serum lipid biomarkers and IMCL. We found that EMCL did not significantly change after intermittent muscle contraction (Figure 2). Schrauwen‐Hinderling et al. (2003) found that EMCL did not change after a cycling exercise of extended duration in both active and non‐active muscles. Although this study supported our findings, several factors related to muscle metabolism differed between the studies, including the age of the participants (young vs. old), their characteristics (highly trained athlete vs. non‐athlete), exercise type (cycling vs. intermittent muscle contraction), and duration (180 vs. 30 min). The role of EMCL in muscle tissue should be investigated in future studies.

We investigated the effect of muscle contraction induced by NMES on IMCL, EMCL, blood lipids, and glucose concurrently. After the 30‐min NMES, we observed that serum TG and glucose levels, which are parameters for metabolic syndrome and diabetes (Table 2), declined significantly. Although these patients were not included in this study, our findings may be applicable in lipid profiling during acute or continuous medical care for patients with metabolic impairments. Further, following NMES, we observed a significant decline in FFAs, but no change in IMCL and EMCL, (Table 2 and Figure 2). When interpreting this finding, it is important to consider the effects of lactate on lipolysis. We observed a significant decline in blood glucose (Table 2), suggesting that glucose contributes to the muscle contraction as an energy source, leading to lactate production; this finding is consistent with that of a previous study (Hamada et al., 2003). Lactate is a key factor in the inhibition of lipolysis (Ahmed et al., 2010). Interestingly, van Loon et al. (2005) found that the decline of IMCL level during exercise was enhanced when lipolysis was inhibited in the adipose tissue. Considering these findings, although our experiment induced increased lactate levels, this increase did not induce inhibition of lipolysis. However, we were unable to determine whether this observation could be because of lacking lactate data. A previous study reported the relationship between IMCL content and insulin resistance (Goodpaster et al., 2001), and in an animal study, acute and chronic lactate infusion was also shown to induce insulin resistance (Vettor et al., 1997). The relationships between changes in IMCL, lipolysis, lactate, and insulin resistance during exercise in humans are still unknown. The nature of these relationships, and their underling mechanisms, are key factors that should be considered in future studies investigating the effects of exercise on FFAs and IMCL.

We observed a significant decline in adiponectin following NMES‐induced intermittent muscle contractions (Table 2). Adiponectin is considered to be one of the cytokines that contributes to the development of insulin sensitivity, and lower levels of adiponectin have been found in patients with obesity and type 2 diabetes (Lihn et al., 2005). Further, adiponectin had a negative relationship with IMCL in the soleus muscles of young patients with type 2 diabetes (Thamer et al., 2002) and obesity (Weiss et al., 2003). Thus, we speculated that adiponectin would decrease if muscle contraction induced a decline in IMCL. However, contrary to our hypothesis, adiponectin significantly decreased after the 30‐min intervention, although the IMCL level did not change (Figure 2 and Table 2). Several studies have investigated the acute effects of exercise on adiponectin levels (Fatouros et al., 2005; Hopps et al., 2011); however, these results were not consistent because of factors affecting exercise‐induced metabolism, such as exercise type and intensity, age, and participant characteristics (e.g., athlete, sedentary, and obese). In interpreting our results, it is important to note that adiponectin in older individuals has features that oppose that of young individuals. Several studies recently observed that older individuals with higher adiponectin levels had higher risk of physical disability and mortality than those with lower adiponectin levels, the so‐called “adiponectin paradox” (Baker et al., 2019). Hence, considering these findings, the decline in adiponectin in our participants might contribute positively to their long‐term health promotion. The underling mechanism of this decline in adiponectin is still not well known; however, Yatagai et al. (2003) proposed that the exercise‐induced decline in adiponectin might be caused by changes in insulin action, fasting glucose levels, and catecholamine activation. We demonstrated a statistically significant change in adiponectin, but this change was small (relative change was approximately 5%) compared with that in FFAs (relative change of FFA was approximately 30%). Further research is required to determine the persistence of this small change, and whether is physiologically and functionally valuable.

Our small sample size was the first limitation of this study. We recruited 24 participants. The number of participants and the minimum number of participants (>5) were determined based on previous studies; several studies have investigated IMCL with under 10 participants (Bucher et al., 2014; Ipavec‐Levasseur et al., 2015; Schrauwen‐Hinderling et al., 2003; Weis et al., 2009; White et al., 2003). Further, we did not include a control group because it was reasonable to speculate that sitting for 30 min would not change muscle metabolism; Krssak et al. (2000) reported that IMCL did not change during a 12‐hour period in the control group (*n* = 3). Unexpectedly, we failed to measure the IMCL and EMCL values in six participants, and they were excluded from this experiment (Figure 1). To confirm the effect of the small sample size, we performed a post hoc ES analysis, as this is an important index supporting the *p*‐value. The post hoc ESs of IMCL, EMCL, serum lipids, and adipocytokine were calculated using *r*. The *r* of IMCL and EMCL was categorized as “small” (<0.10). Therefore, some results should be interpreted with caution due to the possibility of a type II error. To avoid a type II error, large‐scale studies are required in future, such as one examining a community‐dwelling population. Our study was also limited by variations in individual data and methodological precision. IMCL and EMCL levels are affected by several factors, including daily physical activity, exercise habits, nutrition, and calorie intake (Bucher et al., 2014; Haus et al., 2011; van Loon & Goodpaster, 2006). The coefficient of variation (CV) of IMCL was 0.52 and that of EMCL was 0.50, and these values were similar or higher than those obtained in previous studies (Akima et al., 2016; Bucher et al., 2014). We asked our participants to conduct their ordinary daily physical activity and food intake during the intervention; however, it was difficult to control all factors. In addition, methodological precision is associated with the effect of intervention. Previous studies confirmed that muscle lipid levels did not change after the rest, implying the statistical reliability of our measurements (Krssak et al., 2000). However, our findings should be interpreted with consideration of the limitations of individual data variation and methodological precision, as this was a one‐group study.

## 5 CONCLUSION

The purpose of this study was to investigate the effect of muscle contraction induced by NMES on skeletal muscle lipids and serum lipids, including adiponectin, in older individuals. For this purpose, we compared IMCL, EMCL, FFAs, and adiponectin before versus after intermittent muscle contraction induced by NMES (5‐s on‐ and 3‐s off‐duty cycles, for 30 min). FFAs significantly decreased after muscle contraction, although IMCL and EMCL did not change. In addition, we confirmed a significant decline in adiponectin. These findings suggest that FFAs were the energy source utilized during involuntary muscle contraction. This information is important for the development of an effective strategy to prevent and improve insulin resistance in older individuals, a population at high‐risk for metabolic disorders. We plan to perform further comprehensive longitudinal training studies using this muscle contraction technique, not only in healthy older individuals, but also in older individuals with disabilities and patients lacking motor function control, such as those suffering from spinal cord injury, paralysis, and stroke.

## CONFLICT OF INTEREST

The authors have no conflicts to disclose.

## AUTHOR’S CONTRIBUTIONS

The experiments were performed in the Brain & Mind Research Center, Nagoya University, Japan. Akito Yoshiko, Teruhiko Koike, Noriko Tanaka and Hiroshi Akima contributed to the conception and design of the research. Akito Yoshiko, Hisashi Maeda and Hiroshi Akima conducted experiments. Akito Yoshiko performed data analysis and interpreted the results. Akito Yoshiko drafted the manuscript. Akito Yoshiko and Hiroshi Akima edited and revised the manuscript. All authors approved the final version of the manuscript and agree to be accountable for all aspects of the work in ensuring that questions related to the accuracy or integrity of any part of the work are appropriately investigated and resolved. All persons designated as authors qualify for authorship, and all those who qualify for authorship are listed.
